# The Nonsteroidal Anti-Inflammatory Drug Indomethacin Induces Heterogeneity in Lipid Membranes: Potential Implication for Its Diverse Biological Action

**DOI:** 10.1371/journal.pone.0008811

**Published:** 2010-01-21

**Authors:** Yong Zhou, John F. Hancock, Lenard M. Lichtenberger

**Affiliations:** Department of Integrative Biology and Pharmacology, University of Texas Medical School, Houston, Texas, United States of America; University of Helsinki, Finland

## Abstract

**Background:**

The nonsteroidal anti-inflammatory drug (NSAID), indomethacin (Indo), has a large number of divergent biological effects, the molecular mechanism(s) for which have yet to be fully elucidated. Interestingly, Indo is highly amphiphilic and associates strongly with lipid membranes, which influence localization, structure and function of membrane-associating proteins and actively regulate cell signaling events. Thus, it is possible that Indo regulates diverse cell functions by altering micro-environments within the membrane. Here we explored the effect of Indo on the nature of the segregated domains in a mixed model membrane composed of dipalmitoyl phosphatidyl-choline (di16∶0 PC, or DPPC) and dioleoyl phosphatidyl-choline (di18∶1 PC or DOPC) and cholesterol that mimics biomembranes.

**Methodology/Principal Findings:**

Using a series of fluorescent probes in a fluorescence resonance energy transfer (FRET) study, we found that Indo induced separation between gel domains and fluid domains in the mixed model membrane, possibly by enhancing the formation of gel-phase domains. This effect originated from the ability of Indo to specifically target the ordered domains in the mixed membrane. These findings were further confirmed by measuring the ability of Indo to affect the fluidity-dependent fluorescence quenching and the level of detergent resistance of membranes.

**Conclusion/Significance:**

Because the tested lipids are the main lipid constituents in cell membranes, the observed formation of gel phase domains induced by Indo potentially occurs in biomembranes. This marked Indo-induced change in phase behavior potentially alters membrane protein functions, which contribute to the wide variety of biological activities of Indo and other NSAIDs.

## Introduction

Nonsteroidal anti-inflammatory drugs (NSAIDs) are used to treat a wide variety of illnesses and diseases, including inflammation, cancers (colon, gastric, esophageal, pulmonary, prostate, ovarian and breast); cardiovascular disease (myocardial infarction, thrombosis and stroke); diabetes (insulin-resistant and related metabolic syndrome); and diseases of the peripheral and central nervous system (Alzheimer's disease, Parkinson's) [1]. This versatility is attributed to a wide variety of effects of these drugs on cell function. The anti-inflammatory effect of NSAIDs arises from their interaction with enzyme cyclooxygenase (COX), while other biological effects of these drugs are COX-independent, including their effects on tight junctions [2], neutrophil adhesion and transmigration [3], and cell apoptosis and proliferation [4], [5]. Although NSAID-COX interaction has been well characterized, the molecular mechanisms for their COX-independent activities are still not clear. Interestingly, NSAIDs are amphiphilic molecules and interact strongly with lipid membranes [6], [7], [8]. As localization, structure and function of many membrane proteins, enzymes and receptors depend on the cell membrane properties [9], such as fluidity and local curvature, the biological versatility of NSAIDs can potentially originate from their ability to interact with the lipid constituents of membranes.

Indeed, the ability of NSAIDs to associate with lipid membranes is indicated by their high membrane partition coefficients [10], which leads to compromised membrane integrity [6]. Physiologically, NSAIDs increase wettability of the lipid monolayer lining the gastric mucosa and attenuate the barrier properties of the mucosa [8]. Further, the anti-inflammatory agent, salicylate, significantly alters the biomechanical properties of model membranes, such as bending stiffness and pore formation [6]. One of the membrane properties that actively influence localization and function of membrane-associating proteins is fluidity, which is determined by phase behavior of the membrane. Cell membranes are not homogeneous, but contain isolated and dynamic lipid nanoclusters [11]. The formation of these micro-domains under physiological conditions is driven by the immiscibility between lipids with high T_m_, such as fully saturated lipids, and lipids with low T_m_, such as unsaturated lipids. At physiological temperatures, highly hydrophobic and non-polar cholesterol and lipids with high T_m_, usually with fully saturated acyl chains, pack unfavorably with lipids with low T_m_ because, under physiological conditions, lipids with low T_m_ usually contain multiple double bonds and are more polar. This difference in polarity gives rise to immiscibility among different lipid types and induces phase separation and formation of segregated domains. Cholesterol and lipids with high T_m_ are enriched in a liquid-ordered, L_o_, domain, while unsaturated lipids with low T_m_ are enriched in a liquid-disordered, L_d_, domain. These dynamic lipid clusters in L_o_ or L_d_ phases provide platforms for membrane protein aggregation, folding and receptor-ligand binding and affect cell signaling [11], [12]. Although micrometer-sized domain segregation has only been observed in synthetic model membranes, nanometer-sized clusters composed of lipids and proteins have been observed in biological cell membranes using electron microscopy in combination with statistical spatial analysis [11], [12], [13], [14]. Partitioning of the highly amphiphilic NSAID molecules into a membrane has the potential to significantly alter the polarity of some portions of the membrane and hence immiscibility of lipids in the bilayer, and so influence membrane phase behavior and phase separation. The potential change in membrane heterogeneity could lead to changes in localization and function of membrane-associating proteins and enzymes. However, to the best of our knowledge, this possibility has not been explored.

In this study, we examined the effect of a potent NSAID, indomethacin (Indo), on the phase behavior of a mixed model bilayer mimicking biological membranes composed of saturated and unsaturated phosphatidylcholine (PC) with or without cholesterol (Chol). We chose a 3-component membrane composed of 1∶1∶1 mixture of dipalmitoyl phosphatidyl-choline (DPPC, or di-16∶0 PC), dioleoyl phosphatidyl-choline (DOPC, or di-18∶1 PC) and cholesterol because these lipids are main constituents found in epithelial cell plasma membranes [15]. A series of fluorescent probes that favor various phases were utilized in a fluorescence resonance energy transfer (FRET) study, which has been established to be an effective method in characterizing phase separation in membranes [16], [17], [18]. The preference of probes for various membrane phases was determined by measuring the partition coefficients of each probe into bilayers in different phases [16]. These probes include *trans*-parinaric acid (*t*-PnA) (favors gel phase), 1, 2-dipalmitoyl phosphatidyl-ethanolamine-N-(7-nitro-2-1,3-benzoxadiazol-4-yl), or DPPE-NBD (favors L_o_ phase), and 1,2-dioleoyl-phosphatidyl-ethanoloamine-N-(7-nitro-2-1, 3-bendzoxadiazol-4-yl), or DOPE-NBD (favors liquid phase and distributes equally in both L_o_ and L_d_, phases) [16], [19], [20]. We found that Indo significantly enhanced phase separation between gel domains and liquid domains, possibly by inducing the formation of gel-phase domains in the mixed membranes. This was caused by the ability of Indo to specifically target ordered domains. The formation of tightly packed domains was further confirmed by characterizing Indo-induced changes in the fluidity and detergent resistant properties of the mixed model membrane. Because the lipids tested in this study are major constituents in cell membranes, the observed effects of Indo, i.e. formation of gel-phase domains, potentially occur in cell membranes. This effect potentially contributes to the diverse biological effects of Indo and other NSAIDs.

## Results

### Indomethacin Induces Gel-Liquid Phase Separation in DOPC/DPPC/Chol Liposomes

**Figure 1:**
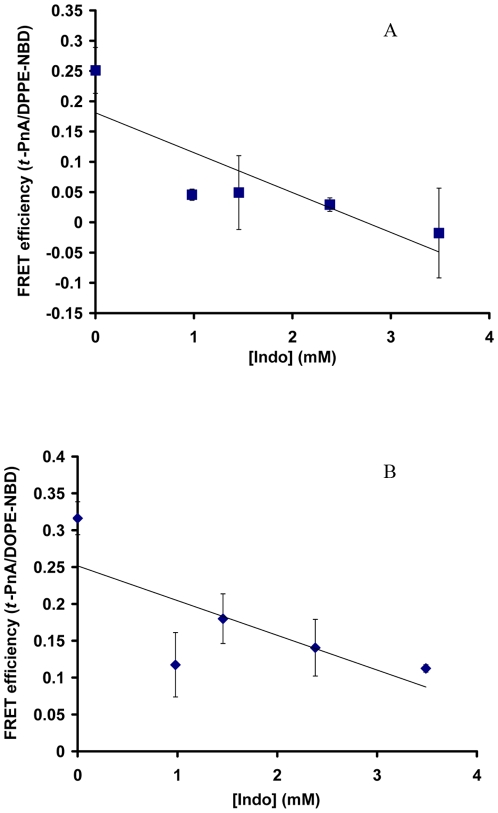
Effect of Indo on FRET efficiency in the mixed membrane (DOPC/DPPC/Chol, 1∶1∶1). Effect of Indo on the efficiency of energy transfer between *t*-PnA (gel phase) and DPPE-NBD (L_o_ phase) (*A*); and between *t*-PnA and DOPE-NBD (liquid phases) (*B*) in the mixed DOPC/DPPC/Chol (1∶1∶1) membrane. The marked decrease in FRET efficiency (p<0.05) indicates significant increase in the distance between the probes and potential separation of gel-phase domains from liquid-phase domains. Trend lines are present to aid the eye of the reader.

Liposomes composed of DOPC/DPPC/Chol (1∶1∶1) were labeled with two probes: *t*-PnA (gel phase) and DPPE-NBD (favors L_o_ phase), where *t*-PnA is the energy donor and NBD is the energy acceptor in a FRET experiment [16]. Without Indo, the energy transfer between the probes was ∼0.25 (Fig. 1a). Indo also induced a dose-dependent decrease in the FRET efficiency to near zero at 3.5 mM Indo (Fig. 1A), suggesting an almost complete separation of the probes and significant segregation of gel domains from L_o_ domains in the mixed membrane. As the original membrane composed of DOPC, DPPC and Chol most likely contains domains in either L_o_ or L_d_ phase, our findings of prominent gel-liquid phase separation suggest that Indo induced the formation of gel-phase domains in the mixed membrane.

To further understand the nature of the liquid phase, we labeled the mixed membrane with *t*-PnA and DOPE-NBD (liquid phases and equally in L_o_ and L_d_ phases). Without Indo, the efficiency of energy transfer between the two probes was approximately 0.32 (Fig. 1B). Addition of Indo induced a dose-dependent decrease in the FRET efficiency, which was ∼0.11 at 3.5 mM Indo, a ∼67% reduction (Fig. 1B). This indicates a marked increase in the distance between the probes and suggests that Indo induced separation between gel and liquid domains.

### Indo Induced Formation of Gel Domains in L_o_ Membrane Composed of DPPC/Chol

**Figure 2:**
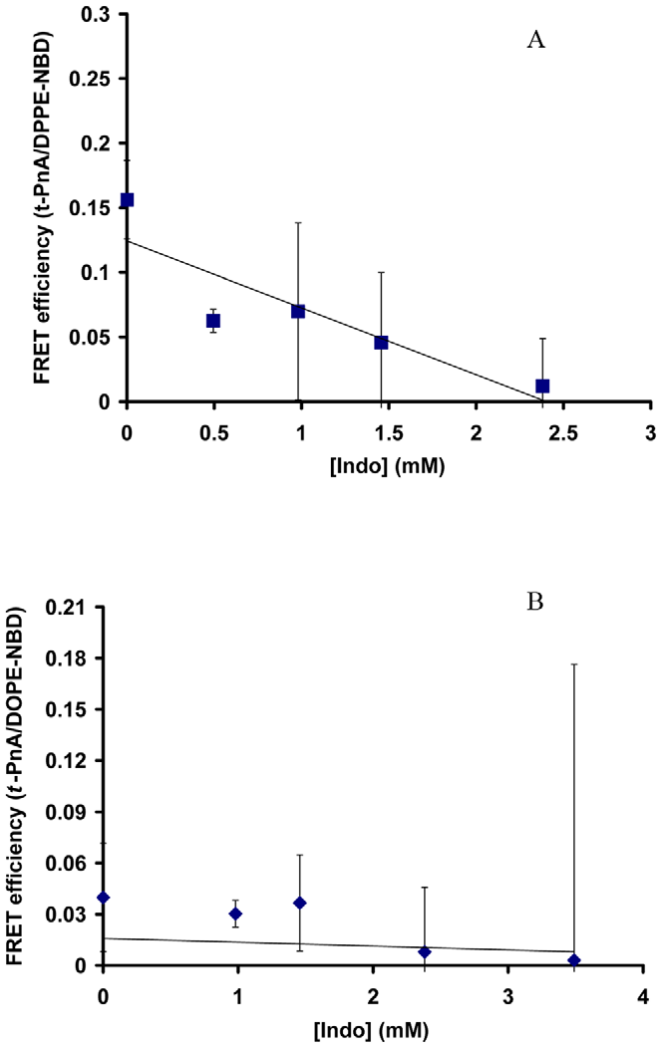
Effect of Indo on FRET efficiency in DPPC/Chol (30 mole%). Effect of Indo on FRET efficiency between *t*-PnA and DPPE-NBD (*A*); and between *t*-PnA and DOPE-NBD (*B*) in DPPC/Chol (30%). The energy transfer between the *t*-PnA and DOPE-NBD was significantly decreased (p<0.05), indicating effective phase separation between gel domains and L_o_ domains. The FRET efficiency between *t*-PnA and DPPE-NBD was not changed significantly (p>0.05) at all Indo concentrations tested.

In an effort to better understand how Indo induces alterations in phase behavior in the mixed DOPC/DPPC/Chol membrane, we wanted to examine the possible effect of Indo on individual components in the mixed membrane in separate experiments. During a possible phase separation in the above-studied mixed membrane (DOPC/DPPC/Chol), DPPC and cholesterol typically aggregate together to form ordered domains in L_o_ phase, while DOPC alone forms disordered domains in L_d_ phase. Thus, we first examined the possible effect of Indo on the L_o_ membrane composed of DPPC and cholesterol (30%). Indo significantly decreased the energy transfer between *t*-PnA (gel phase) and DPPE-NBD (L_o_ phase) from ∼0.16 without Indo to ∼0.01 in 2.4 mM Indo (Fig. 2A), indicating a nearly complete separation between the two probes and further suggesting that Indo induced significant segregation of gel phase domains from L_o_ domains. The energy transfer between *t*-PnA and DOPE-NBD was minimally changed by Indo (Fig. 2B), possibly because the FRET efficiency between the probes was extremely low (∼0.04) even without Indo.

### Indo Had No Effect on the L_d_ Membrane of DOPC

**Figure 3:**
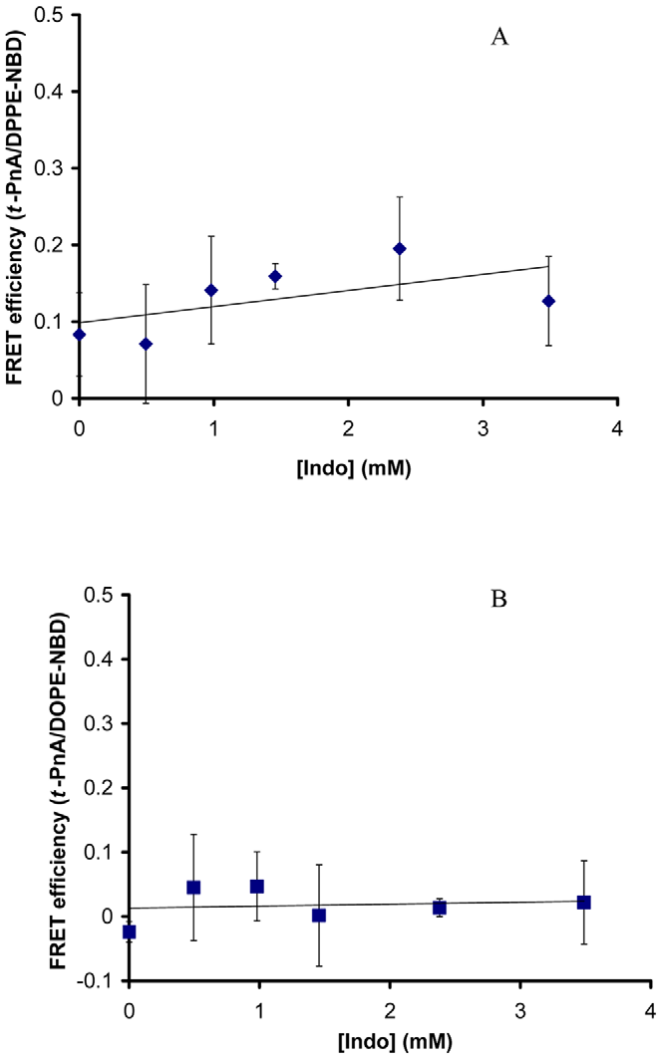
Effect of Indo on FRET efficiency in 100% DOPC. Effect of Indo on FRET efficiency between *t*-PnA and DPPE-NBD (*A*); and between *t*-PnA and DOPE-NBD (*B*) incorporated into pure DOPC bilayer. Indo at all concentrations had no effect on FRET efficiency between probes either FRET pair (p>0.05).

In the mixed DOPC/DPPC/Chol membrane, DOPC alone forms the L_d_ domains. We then performed FRET experiments and found that Indo at concentrations tested did not alter the energy transfer between either *t*-PnA and DPPE-NBD or *t*-PnA and DOPE-NBD in pure DOPC membrane (Fig. 3A and B). This suggests that Indo has no observable effect on the phase behavior of DOPC and that the Indo-induced gel/liquid phase separation in the mixed DOPC/DPPC/Chol did not originate from interactions between Indo and DOPC. We note that Indo at 2.4 mM induced a numerical increase in FRET efficiency between *t*-PnA and DPPE-NBD in DOPC, although not statistically significant. As an increase in FRET efficiency between membrane probes suggests closer association of the probes and more homogeneous mixing of the material within the membrane and DOPC was already in a homogeneous L_d_ phase without Indo, this potential increase induced by Indo is consistent with our hypothesis that Indo has no effect on the phase behavior of DOPC.

Experiments on DOPC liposomes also indicated that Indo-induced changes in FRET efficiency detected in mixed membranes described in previous sections are *not* caused by a general disruption of the entire membrane. Compared with mixed membranes containing highly hydrophobic disaturated DPPC and membrane-stabilizing cholesterol, pure DOPC membranes should be more prone to amphiphilic attack. Yet, we found no change in FRET efficiency in DOPC membrane exposed to the NSAID, suggesting that possible loosened packing is not the source for Indo-induced changes in FRET efficiency observed in the mixed membranes. Thus, phase separation should be the likely origin for Indo-induced changes in energy transfer found in previous sections. Further, these experiments, which demonstrate that Indo does not induce a change in FRET efficiency in DOPC membranes also demonstrated that Indo did not directly interact with the probes and did not affect their inherent fluorescent properties.

### Detergent Resistant Membrane Level Is Altered by Indo

**Figure 4:**
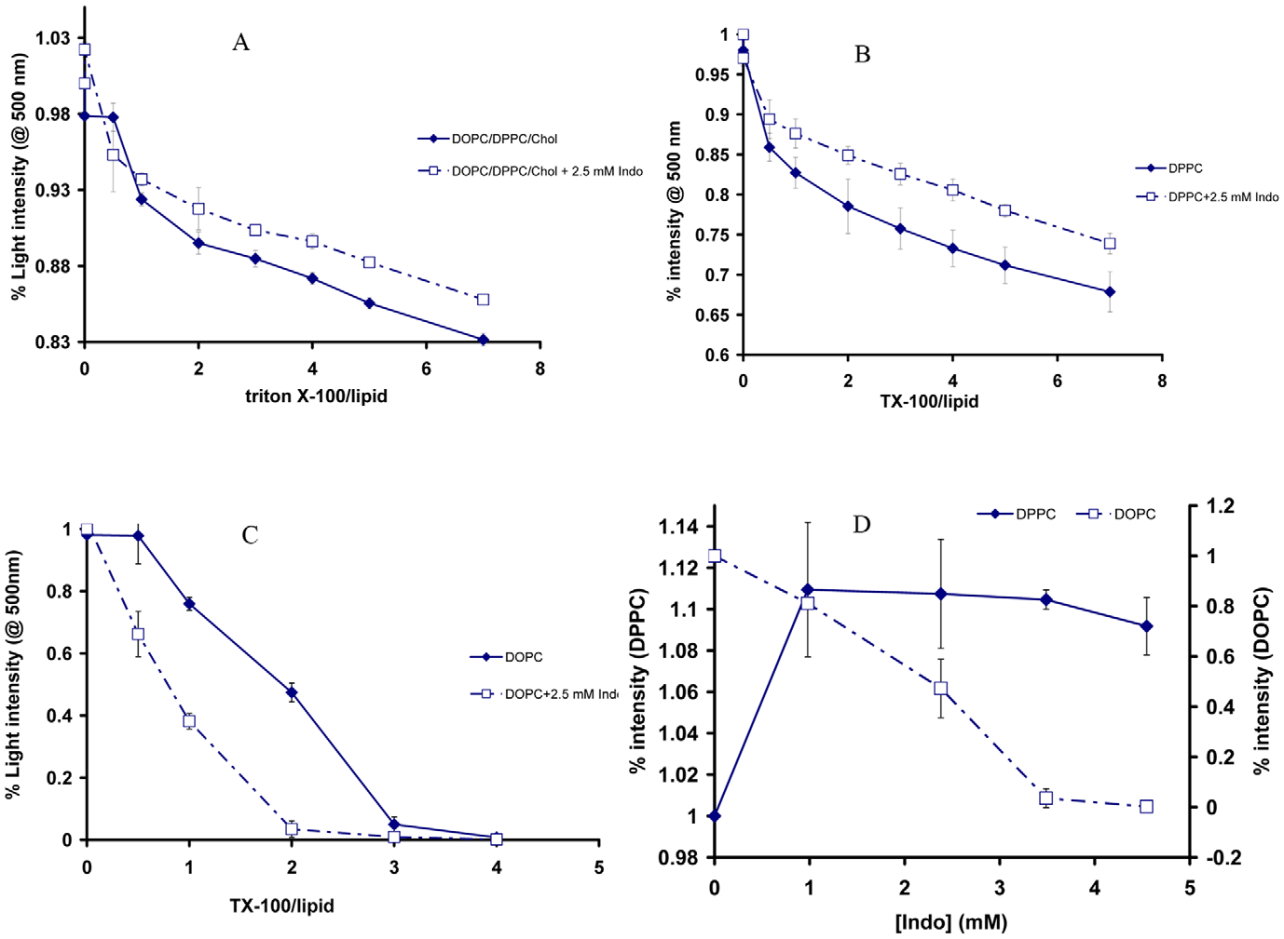
The ability of Indo to influence detergent resistant membrane against detergent effects of triton X-100. *A*) Higher turbidity measurements were obtained in DOPC/DPPC/Chol (1∶1∶1) membrane exposed to 2.5 mM Indo, indicating a better ability of the mixed membrane exposed to Indo to resist the detergent effect of triton X-100 (p<0.05). *B*) The ability of pure DPPC to resist triton X-100 solubilization was also increased significantly (p<0.05) by 2.5 mM Indo. *C*) The ability of pure DOPC to resist the detergent effect of triton X-100 was significantly *diminished* by 2.5 mM Indo (p<0.05). *D*) The contrasting effect of changing Indo concentrations on detergent resistance of DPPC (*closed diamonds*) and DOPC (*open squares*) against triton X-100. Different levels of triton X-100 were used for each lipid type to achieve optimal solubilization: 30 µl for DPPC or 9 µl for DOPC (See Method section).

The above FRET experiments suggest that Indo induced gel/liquid phase separation may be caused by the formation of gel domains that segregate from the existing liquid domains. Alternatively, the same FRET data could be explained by the possibility that Indo could induce the formation of liquid domains that segregate from the existing gel domains. Our data suggests the former because the original mixed membrane without Indo is primarily composed of L_o_ domains (DPPC/Chol) and L_d_ domains (DOPC). To test this possibility, the ability of Indo to alter detergent resistant membrane level of the mixed membrane was evaluated because the ability to resist detergent extraction is a characteristic of formation of dense gel/ordered domains in the membrane. The solubilization of DOPC/DPPC/Chol (1∶1∶1) liposomes was assayed by measuring lipid turbidity at 500 nm when exposed to changing concentrations of triton X-100. All experiments were performed at room temperature. We found that the presence of 2.5 mM Indo caused a significant shift of the turbidity curve to the right in the presence of triton X-100 (Fig. 4A), hence the NSAID protected domain lipids from being solubilized, further suggesting that Indo induced formation of highly ordered and tightly packed domains in DOPC/DPPC/Chol liposomes, in agreement with our FRET study.

To better understand which lipids were protected by Indo from triton X-100, we tested the effect of Indo on the detergent resistant ability of individual lipids, DPPC or DOPC. For DPPC, Indo caused less lipid solubilization, a similar effect as observed in the mixed membrane (Fig. 4B). However, Indo increased solubilization of pure DOPC bilayers (Fig. 4C), opposite of the effect of the NSAID on the mixed membrane or pure DPPC. Additionally, we tested the ability of increasing Indo concentrations to alter the detergent resistance of DPPC or DOPC exposed to a fixed triton X-100 level. Again, as depicted in Fig. 4D, a contrasting effect Indo was found: increasing concentrations of Indo induced higher turbidity measurements in DPPC suspensions (*closed diamonds*) and a significant decrease in turbidity of DOPC suspension (*open squares*). This suggests that Indo induced less lipid solublization and led to an increased detergent resistance for DPPC and more solubilization of DOPC. This set of experiments further suggests that the Indo induced tighter packing and enhanced stabilization of the ordered domains containing DPPC. The enhanced solubilization of DOPC suggests better mixing among Indo, triton X-100 and DOPC lipids.

### Indo Prevents Fluorescent Quenching of DPH in DOPC/DPPC/Chol Membranes

**Figure 5:**
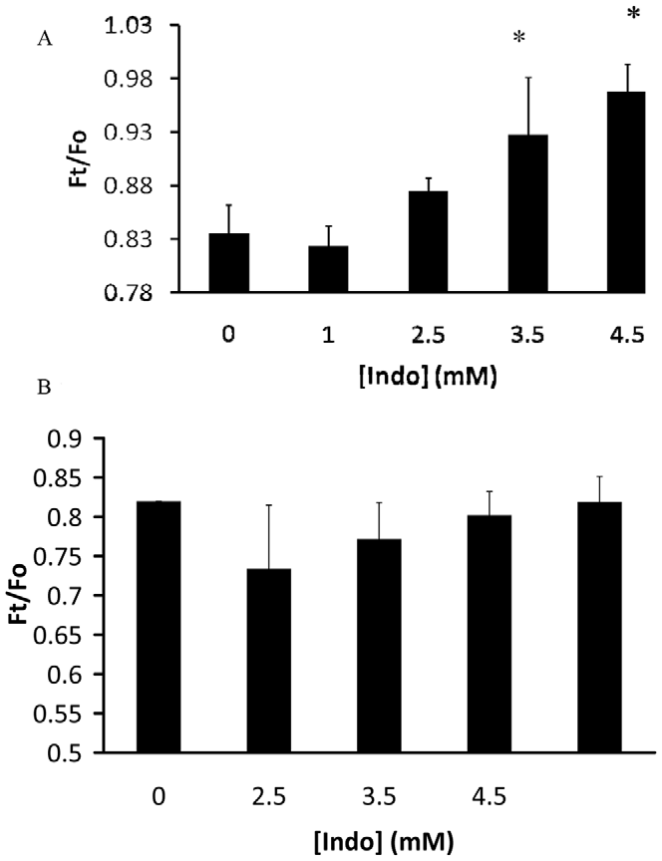
Effect of Indo on fluidity-dependent fluorescent quenching in membranes. Effect of Indo on the ability of TEMPO to quench DPH fluorescent intensity in mixed DOPC/DPPC/Chol (1∶1∶1) (*A*) and pure DOPC (*B*) membranes. Approximately 5 mg of lipids at 2.5 mg/ml were exposed to 2 mM TEMPO in each experiment at 24°C. Indo led to statistically significant increase in values of *F_o_/F_t_* for the mixed membrane (p<0.05), while having no detectable effect on *F_o_/F_t_* values for DOPC bilayer. This indicates that Indo had no effect on the quenching efficiency of TEMPO in DOPC, but significantly diminished the quenching efficiency of TEMPO in the mixed membrane. Since quenching is achieved by partitioning of hydrophilic TEMPO into the fluid domains, the observed diminished quenching suggest the formation of tightly packed membrane patches that exclude TEMPO.

To further confirm the ability of Indo to induce gel domains in the mixed membrane, we characterized the effect of Indo on the ability of a fluorescent quencher TEMPO to quench the intensity of a hydrophobic probe DPH. This experiment utilizes two unique properties: DPH partitions into all membrane phases and the hydrophilic TEMPO only partitions into more disordered fluid phases [21]. A parameter of *F_t_*/*F_o_* (*F_t_* is DPH intensity in the presence of the quencher TEMPO while *F_o_* is DPH intensity without TEMPO) indicates the proportion of the densely packed gel-ordered domains, hence phase behavior of the tested membrane [21]. In Fig. 5A, the *F_t_*/*F_o_* value of DPH in DOPC/DPPC/Chol (1∶1∶1) was ∼0.8, in a good agreement with findings in the literature [21]. Larger *F_t_*/*F_o_* values of DPH were obtained in the mixed membrane exposed to various concentrations of Indo (Fig. 5A). As the values of *F_t_*/*F_o_* are inversely related to the quenching efficiency of TEMPO, which is dependent upon the packing density of the membrane, our data suggest that Indo induced formation of dense, possibly gel phase, domains that prevented partitioning of TEMPO and the subsequent quenching of DPH in the mixed membrane. This finding supports the findings in our FRET and detergent resistant membrane experiments. We then found that Indo had no detectable effect on the ability of TEMPO to quench DPH in DOPC liposomes (Fig. 5B). This result also suggests that Indo did not affect the phase behavior of the highly disordered DOPC membrane, as suggested by our FRET and detergent resistant membrane experiments. The minimal effect of Indo on TEMPO quenching efficiency of DPH incorporated in DOPC membranes also shows that Indo did not directly interact with TEMPO.

## Discussion

In this study, we aimed to examine the ability of an NSAID, Indomethacin (Indo), to alter membrane phase behavior. Such an possible effect could explain a wide variety of biological effects of Indo, and possibly other NSAIDs and amphiphilic drugs, because membrane phase behavior determines localization, structure and function of membrane proteins. We utilized a series of FRET studies to characterize the properties of segregated domains in the mixed DOPC/DPPC/Chol (1∶1∶1) membrane exposed to Indo. The FRET pairs include: *t*-PnA (gel phase) and DOPE-NBD (liquid phase in general) and *t*-PnA and DPPE-NBD (L_o_ phase). Interestingly, *t*-PnA partitions equally into both L_o_ and L_d_ domains when there is no gel domain [16]. As all probes used in this study partition into liquid phases and only *t*-PnA intercalates into gel domains, the only time that there would be a change in the FRET efficiency is when there is phase separation between gel domains and liquid phases in the membrane. Since the mixed membrane without Indo primarily contains various liquid domains in L_o_ and L_d_ phases, our data suggest that Indo potentially induced significant formation of gel-phase domains, which was further confirmed by detergent resistant membrane and fluidity-dependent fluorescence quenching experiments.

Interestingly, we found that Indo specifically targets ordered domains (DPPC/Chol) and has no effect on the disordered fluid domains (DOPC). Indo has also been found to induce heterogeneity in single-component DPPC or DMPC bilayers [10], [22]. Our observation is in-line with the concept of Indo enhancing immiscibility in the membrane. In a pure membrane in gel phase, such as single-component membranes composed of lipids with high T_m_ (e.g. DPPC), immiscibility between Indo and lipids excludes Indo molecules from the gel-phase lipids and causes the formation of highly fluid domains. In mixed bilayers mimicking biological membranes, such as DOPC/DPPC/Chol membrane, as was tested in this study, immiscibility between hydrophobic DPPC/Chol and more polar DOPC drives the formation of segregated domains. Intercalation of highly polar Indo into the membrane will undoubtedly enhance the polarity of the area enriched with Indo molecules. This increase in polarity potentially drives further segregation of DPPC and cholesterol from L_d_ membranes because DPPC and cholesterol packs unfavorably with the highly polar Indo molecules. The enhanced segregation of DPPC/Chol would lead to tighter packing of DPPC and cholesterol molecules and an increase in the ordering of lipids in L_o_ domains and so promote the formation of gel-phase clusters. The exact physical nature of the gel domains induced by Indo is, however, not clear and will be examined in more detail in later studies.

The Indo-induced phase separation was supported by our detergent resistant membrane experiments, which demonstrated that Indo enhanced the ability of the mixed membrane to resist solubilization by triton X-100, an indication of the formation of more tightly packed membrane domains. As previously indicated, detergents have been established to alter membrane properties. This is especially a problem when attempting to isolate lipid domains and domain-associating proteins in biological cells. Detergent treatment enhances phase separation and potentially introduces artificially induced domain-associating lipids and proteins [11], [23], [24]. Because these type of experiments cannot be performed on biological membranes in the absence of detergents, one simply cannot distinguish between true domain-associating lipids and proteins and those created by the detergents (the artifacts) [11], [23], [24]. Hence, this technique, when used in biological cells, lacks proper control and the data interpretation should be treated with caution [11], [23], [24]. However, in synthetic systems, this problem does not exist. We have performed proper controls for this experiment in synthetic systems and indicated how Indo alters lipid turbidity without triton X-100. A comparison between triton treatment with and without Indo clearly indicates that Indo induces increased stability of membranes containing DPPC and cholesterol and the opposite was found in DOPC in the presence of triton X-100. The observed increase in detergent resistance conferred by Indo to DPPC and DPPC/DOPC/Chol vesicles, therefore cannot be simply attributed to a detergent effect, as vesicles composed of DOPC alone clearly become less detergent resistant in the presence of both Indo and triton X-100. The presence of these gel-like domains in the mixed membrane was also confirmed by TEMPO quenching experiments, where the ability of the hydrophilic TEMPO to quench DPH intensity was prevented by Indo. This suggests that Indo induced the formation of lipid domains that exclude TEMPO molecules. Partitioning of amphiphilic Indo molecules into a similarly polar and fluid pure DOPC bilayer does not significantly alter the inter-lipid interactions, inducing limited immiscibility, which was shown by the FRET experiments. Our detergent resistant membrane experiments also demonstrated that Indo enhanced solubilization of DOPC lipids by triton X-100, which suggests better mixing among 3 components. Thus, Indo has little effect on the phase behavior of the DOPC membrane.

Here, we demonstrated that Indo induced formation of gel-phase domains in synthetic mixed DOPC/DPPC/Chol membranes. This potentially may have marked effects on cell functions. Although cell membranes are mostly fluid, highly ordered and segregated nanoclusters composed of lipids with high T_m_ and cholesterol are present in cell plasma membranes and actively regulate protein localization, protein-protein association and receptor-ligand binding [12], [25], [26]. Since DPPC and cholesterol are major components in these nanoclusters in biomembranes, our finding suggests that, in cell membranes, Indo specifically affects these clusters and induces changes in phase behavior in these areas. Our data point to the possibility that Indo has the ability to influence the micro-environment of these clusters and potentially alters their interaction with membrane-associating proteins. The concentration of Indo in a human gastric juice can reach as high as 2 to 10 mM and the biophysical properties of phase separation studied in synthetic membranes provide the foundation for our understanding of lateral heterogeneity of biological cell membrane. In this context, an effect of Indo on cholesterol-dependent nanoclusters found in our study using synthetic systems can reasonably be extrapolated to *in vitro* and *in vivo* conditions. We therefore propose that Indo-induced changes in membrane heterogeneity could potentially occur in biological membranes and that this effect is likely to lead to changes in protein aggregation/localization and eventually cell function.

### Conclusions

We found that the amphiphilic drug indomethacin enhances immiscibility of saturated and unsaturated lipids and induces the formation of gel-phase domains in mixed model membrane bilayers. Because the tested lipids comprise the main constituents of biological membranes, we speculate that a similar effect also occurs in cell membranes and that this NSAID effect alters the membrane micro-environment with concomitant changes on protein localization and function. The observed effect of Indo on membrane phase behavior potentially contributes to the wide variety of biological activities of Indo, and related other NSAIDs and amphiphilic drugs.

## Materials and Methods

### Materials

The phospholipids, dipalmitoyl phosphatidyl-choline (DPPC) and dioleoyl phosphatidyl-choline (DOPC) were purchased from Avanti Polar Lipids, Inc (Alabaster, AL, USA). Lipids were dissolved in chloroform and stored at −20°C under nitrogen. Cholesterol (Chol), 1,6-diphenyl-1,3,5-hexatriene (DPH) and quencher, 2,2,6,6-tetramethylpiperdine-1-oxyl (TEMPO) were purchased from Sigma-Aldrich (St. Louis, MO, USA). Fluorescent probes dipalmitoyl phosphatidylethanolamine-7-nitrobenz-2-oxa-l,3-diazol-4-yl (DPPE-NBD), and dioleoyl phosphatidylethanolamine-NBD (DOPE-NBD) were also purchased from Avanti Polar Lipids. Gel-phase probe, *trans*-parinaric acid (*t*-PnA) was purchased from Cayman Chemicals (Ann Arbor, MI, USA).

### Methods

#### Large unilamellar vesicle (LUV) formation

Approximately 20 mg PC lipids in chloroform was dried under nitrogen gas and then exposed to vacuum overnight to completely evaporate chloroform. The lipid film was rehydrated with ∼8 ml of phosphate buffer solution (PBS) at pH 7.4 and incubated for 30 minutes at 66°C, a temperature well above the highest melting temperature in the lipid mixture (T_m_ of DPPC is ∼41°C) under nitrogen gas. The pure DOPC (T_m_ is −20°C), was incubated at 24°C. The lipid suspension was then extruded across a polycarbonate filter with 100 nm pores to form large unilamellar vesicles (LUVs) in a Mini-extruder set-up (Avanti Polar Lipids, Inc) at appropriate temperatures indicated above. The average size of vesicles was verified to be ∼120 nm by dynamic light scattering (data not shown). In all experiments described below, liposomes were incubated for 30 minutes in PBS buffer containing various concentrations of Indo before measurements.

#### Fluorescence resonance energy transfer (FRET)

The technique of using FRET to characterize phase separation in LUVs has been well developed [16], [17], [18]. In our experiments, liposomes composed of DOPC/DPPC/Chol (1∶1∶1) were labeled with various combinations of probes: *t*-PnA and DOPE-NBD or *t*-PnA and DPPE-NBD. LUVs containing 0.5 mole% of either two probes or only the donor probe were formed according to the procedure listed above. Fluorescence intensity of the donor in the presence or absence of the acceptor was measured at room temperature by using QuantaMaster UV/VIS spectrofluorometer (Photon Technology International, Inc. Birmingham, New Jersey). The efficiency of energy transfer, *E*, was calculated using equation: *E* = (*I_D_-I_D-A_*)/*I_D_*, where *I_D_* is the intensity of the donor in sample containing only donor probe and *I_D-A_* is the intensity of the donor in sample containing both donor and acceptor probes.

#### Detergent resistant membrane level

Liposomes composed of DOPC/DPPC/Chol, or single-component of DPPC or DOPC, were prepared as described above. Turbidity of liposome samples exposed to triton X-100 with or without Indo was measured as absorbance at 500 nm at room temperature. Measurements were obtained in Genesys 10UV spectrophotometer (Thermo Scientific, Waltham, MA) with a 1 cm path-length cell. Samples were occasionally stirred to ensure uniform mixing. The turbidity measurements were normalized against the control turbidity value of lipid suspension before triton X-100 and Indo treatment for experiments testing effect of various doses of triton X-100 on lipid turbidity. For experiments examining effect of Indo dose effect, data were normalized against turbidity measurements of lipids initially exposed to fixed amount of triton X-100 but without Indo. Different levels of triton X-100 were used for each lipid type to achieve optimal solubilization: 30 µl for DPPC or 9 µl for DOPC. The rational for the two concentrations of triton X-100 is that 9 ml would be too low to induce detectable solubilization of DPPC, and alternatively 30 ml triton X-100 would be too high for dose-dependent solubilization of DOPC. Characterization of the potential effect of Indo would be impossible in either case.

#### Fluidity-dependent fluorescent quenching

To test formation of tightly packed domains and changes in membrane fluidity, we utilized a fluorescence technique that involves quenching of a membrane probe DPH by a quencher 2,2,6,6-tetramethylpiperdine-1-oxyl, TEMPO [21]. Model liposomes were generated containing DOPC/DPPC/Chol (1∶1∶1) along with 0.5 mole% DPH. For each experiment, 12 µl of 350 mM TEMPO in ethanol was added in a quartz cuvette containing 2 ml lipid suspension while 12 µl of pure ethanol was added in a second cuvette also containing 2 ml of the same lipid suspension as a control to account for any possible effect of ethanol on DPH florescence intensity. The DPH fluorescence intensity after TEMPO exposure (F_t_), along with that of a control sample without TEMPO (F_o_), was measured by using the QuantaMaster UV/VIS spectrofluorometer.
