# Correction: The Nonsteroidal Anti-Inflammatory Drug Indomethacin Induces Heterogeneity in Lipid Membranes: Potential Implication for Its Diverse Biological Action

**DOI:** 10.1371/annotation/b3b1ad62-b95a-4c99-8885-806ef66347df

**Published:** 2010-03-23

**Authors:** Yong Zhou, John F. Hancock, Lenard M. Lichtenberger

Some potential competing interests were not declared for Dr. Lichtenberger. The authors apologize for this omission and would like to disclose the following information:

Dr. Lichtenberger is founder and has equity in PLx Pharma, a start-up company that is developing phospholipid-conjugated nonsteroidal anti-inflammatory drugs (NSAIDs). Dr. Lichtenberger has filed patent applications in relation to NSAID-phospholipid compositions and methods for their preparation. The study was not funded by PLx Pharma and the company does not currently develop any products related to the results described in the article.

